# *DOCK2* Mutation and Recurrent Hemophagocytic Lymphohistiocytosis

**DOI:** 10.3390/life13020434

**Published:** 2023-02-03

**Authors:** Daniel D. Reiff, Mingce Zhang, Randy Q. Cron

**Affiliations:** Department of Pediatrics, Division of Rheumatology, University of Alabama at Birmingham, Birmingham, AL 35233, USA

**Keywords:** hemophagocytic lymphohistiocytosis, inflammation, natural killer cell, macrophage, cytolysis, degranulation

## Abstract

Hemophagocytic lymphohistiocytosis (HLH) is a syndrome resulting from uncontrolled hyper-inflammation, excessive immune system activation, and elevated levels of inflammatory cytokines. HLH can be caused by the inability to downregulate activated macrophages by natural killer (NK) and CD8 cytotoxic T cells through a process reliant on perforin and granzyme B to initiate apoptosis. Homozygous genetic mutations in this process result in primary HLH (pHLH), a disorder that can lead to multi-system organ failure and death in infancy. Heterozygous, dominant-negative, or monoallelic hypomorphic mutations in these same genes can cause a similar syndrome in the presence of an immune trigger, leading to secondary HLH (sHLH). A genetic mutation in a potential novel HLH-associated gene, dedicator of cytokinesis 2 (*DOCK2)*, was identified in a patient with recurrent episodes of sHLH and hyperinflammation in the setting of frequent central line infections. He required baseline immune suppression for the prevention of sHLH, with increased anti-cytokine therapies and corticosteroids in response to flares and infections. Using a foamy-virus approach, the patient’s *DOCK2* mutation and wild-type (WT) control *DOCK2* cDNA were separately transduced into a human NK-92 cell line. The NK-cell populations were stimulated with NK-sensitive K562 erythroleukemia target cells in vitro and degranulation and cytolysis were measured using CD107a expression and live/dead fixable cell dead reagent, respectively. Compared to WT, the patient’s *DOCK2* mutation was found to cause significantly decreased NK cell function, degranulation, and cytotoxicity. This study speaks to the importance of *DOCK2* and similar genes in the pathogenesis of sHLH, with implications for its diagnosis and treatment.

## 1. Introduction

Hemophagocytic lymphohistiocytosis (HLH) is a syndrome of disordered immune system function and regulation, resulting in hyperinflammation and elevated levels of pro-inflammatory cytokines. In response to an immune trigger, activated macrophages drive inflammation, secrete inflammatory cytokines, and phagocytose foreign antigens. However, when left unchecked, activated macrophages can cause tissue damage, hypotension, and organ failure. Natural killer (NK) cells and cytotoxic T lymphocytes function to eliminate activated macrophages, maintain a level of homeostasis, and prevent hyperinflammatory damage. The cytotoxic killing of activated macrophages occurs through a perforin and granzyme B dependent pathway, where a pore is created in the macrophage membrane and apoptotic granules are delivered to initiate cell death [1]. This pathway involves a multitude of genes involved in lysosomal packaging, trafficking, docking, fusion, pore creation, and delivery of cytotoxic granules [2]. Homozygous mutations in any one of these genes can cause ineffective killing of activated macrophages and result in unchecked inflammation. Primary HLH (pHLH) is a result of such homozygous mutations and absent immune regulation, leading to severe inflammation, multi-system organ failure, and frequently death in infancy and early childhood. Heterozygous, monoallelic hypomorphic, or dominant-negative mutations in these same genes can also cause similar hyperinflammatory effects, termed secondary HLH (sHLH). sHLH results from diminished, but not absent, immune regulation in conjunction with an immune trigger, such as an infection, malignancy, or rheumatic disease [3].

The dedicator of the cytokinesis (DOCK) family of proteins is an important intracellular signaling molecule involved in multiple pathways of immune system activation and function. DOCK proteins have been implicated in lymphocyte migration, cytoskeleton formation, differentiation of T cells, immunodeficiency syndromes, release of type 1 interferons, and many other important functions [4,5]. Specific to HLH pathogenesis, homozygous deficiency in certain DOCK2 proteins has been shown to result in impaired degranulation and release of perforin and granzyme B, leading to the uncontrolled proliferation of activated macrophages typified in p- and sHLH [6,7]. In the following report, we describe a patient suffering from recurrent bacterial infections in the setting of an implanted central line, many complicated by bouts of hyperinflammation consistent with sHLH. He was found to have a rare (GnomAD_exome 0.01%) mutation in *DOCK2*, a gene encoding a DOCK protein with important immune regulation functions. His mutation was transduced in vitro into a human NK-92 cell line and found to result in impaired NK cell degranulation and target cell lysis as compared to wild-type (WT) *DOCK2*. This report provides support of a potential role for *DOCK2* genetic defects contributing to sHLH.

## 2. Materials and Methods

### 2.1. Patient Data

The patient’s electronic medical record was reviewed for hospitalizations, outpatient care, therapeutics, and pertinent laboratory values. All information was de-identified following an Institutional Review Board approved protocol. The patient’s *DOCK2* mutation was identified via a commercially available (Invitae, San Francisco, CA, USA) genetic panel screening for pHLH gene variants and other genetic immunodeficiencies. This study using de-identified patient data was conducted using the UAB Institutional Review Board approved protocol, number 120907003.

### 2.2. DOCK2 Wild-Type and Mutant DNA Constructs

Wild-type (WT) *DOCK2* copy DNA (cDNA) was generated from healthy donor peripheral blood mononuclear cells (PBMCs) using reverse transcription (ThermoFisher, Waltham, MA, USA). The WT cDNA sequence was confirmed using Sanger DNA sequence analysis. A human foamy virus (FV) expression system was utilized to insert WT *DOCK2* cDNA into a plasmid vector as previously described [8]. Mutant *DOCK2* cDNA was generated via site-directed mutagenesis, as previously described, based on the WT plasmid and confirmed with Sanger sequencing [9].

### 2.3. Viral Preparation and Transduction

HK293T cells were transfected with WT and mutant *DOCK2* plasmids using FuGene HD (Promega, Madison, WI, USA). Supernatants were harvested after 72 h and concentrated with Lenti-X concentrator reagent (Takara, Kusatsu, Japan). Human NK-92 cell populations were separately infected with either WT- or mutant *DOCK2*-expressing FV and confirmed by flow cytometry for co-expressed green fluorescent protein (GFP) [8]. Transfected NK-92 cells were sorted based on GFP expression, and >98% of both WT- and mutant *DOCK2*-expressing NK-92 cells expressed GFP when studied.

### 2.4. Degranulation and Cytotoxicity Assays

NK-sensitive K562 erythroleukemia cells (provided by Dr. Olaf Kutch, Department of Microbiology, University of Alabama at Birmingham, Birmingham, AL, USA) and FV-infected NK-92 cells were co-incubated for 2–4 h, harvested, and stained with anti-CD56 (NK cell marker, Pacific Blue, Biolegend, San Diego, CA, USA). To study degranulation, the cells were incubated with fluorescein-conjugated anti-CD107a (LAMP1) antibodies (Allophycocyanin, Biolegend, San Diego, CA, USA). For cytotoxicity analysis, cells were stained with live/dead fixable cell dead reagent (Invitrogen, Waltham, MA, USA). In both cases, cells were analyzed by flow cytometry using FlowJo 10.2 software (FlowJo, Ashland, OR, USA) [8]. Statistical analyses were performed with GraphPad Prism 6 (GraphPad Software, La Jolla, CA, USA) software. Two-way ANOVA analysis was used to calculate p values (α = 0.05) for the NK-92 cell cytotoxicity assays [10].

## 3. Clinical History

This black male child was born at 33 weeks via twin gestation with a neonatal course complicated by necrotizing enterocolitis requiring significant bowel resection. After multiple surgeries and complications, including volvulus, he currently has 9% of his small bowel and his rectum remaining, with complete resection of his large intestine. Due to short-gut physiology, he has required parenteral nutrition via an implanted central line from very early in life. At age seven, he had his first episode of hyperinflammation in the setting of fever, emesis, and a negative infectious work-up. During this initial episode, he experienced pancytopenia, liver enzyme elevation, hyperferritinemia, and elevated LDH meeting HLH-2004 criteria [11]. Rheumatology was consulted regarding concern for HLH, and the patient improved substantially with three days of pulse-dose (30 mg/kg/dose) methylprednisolone and initiation of anakinra (recombinant human IL-1 receptor antagonist) via subcutaneous injection [12]. An HLH-specific work-up showed elevated sCD163 (a macrophage-specific scavenger receptor used as a biomarker for HLH) and soluble interleukin 2 receptor-α (sIL-2Ra or sCD25), but a 21-gene HLH panel (Supplemental Table S1) was negative for mutations in known HLH genes [13,14]. Since his initial episode of HLH, he has been hospitalized ten additional times over the last 5 years for various bloodstream infections secondary to his implanted central line, all of which have resulted in varying degrees of HLH flare, typified by fever, pancytopenia, elevated liver enzymes, and high ferritin levels. Initial and subsequent flares are shown below in Table 1 and Table 2.

During his admission at ten years of age for *Bacillus licheniformis* bacteremia, perforin and granzyme expression was noted to be elevated in his NK cells with perforin mean channel fluorescence (MCF) at 191% (normal 98–181%) and granzyme B MCF 1146% (152–835%) [15]. At his next hospitalization at age ten, he was found to have decreased CD107a expression with CD107a positive NK cells at 8% (normal 11–35%) and MCF of 183 (207–378). An immunodeficiency genetic panel during that same hospitalization revealed twelve variants of unknown significance, but only one in a gene potentially involved in HLH pathogenesis via disruption of NK cell function (Table 3). A rare (GnomAD_exome 0.01%) heterozygous missense variant in the *DOCK2* gene c.1334A>G (p.Asn445Ser) was noted, which was thought to be involved in his recurrent bouts of HLH.

Since his initial episode of hyperinflammation, this patient has remained on anakinra at a baseline of 100 mg twice daily via subcutaneous injection. In between flares of disease, his laboratory values, with the exception of baseline diminished NK cell cytolytic function and degranulation, improve to normal and are closely monitored in the setting of chronic total parenteral nutrition [16]. When inflammation flares in the setting of infection, or with unknown trigger, anakinra is increased to 100 mg 2–3 times daily and oral vs. intravenous corticosteroids are added if needed. This regimen has been clinically effective in improving blood counts, decreasing hyperferritinemia, and resolving liver enzyme elevation in the acute setting, with corticosteroids and anakinra weaned slowly in the outpatient setting. Additional family education on safe and sterile access to his central line has diminished hospitalizations for sHLH.

## 4. Results

### DOCK2 Mutation (p.Asn445Ser) Decreases NK Cell Degranulation and Cell Lysis

To further explore the effect of this patient’s *DOCK2* on his recurrent episodes of hyperinflammation, WT *DOCK2* and *DOCK2* c.1334A>G (p.Asn445Ser) mutant cDNA were independently expressed into a human NK cell line NK-92. NK cell function was assessed through analysis of NK cell degranulation and cytotoxicity as detailed in Methods. 

**Figure 1:**
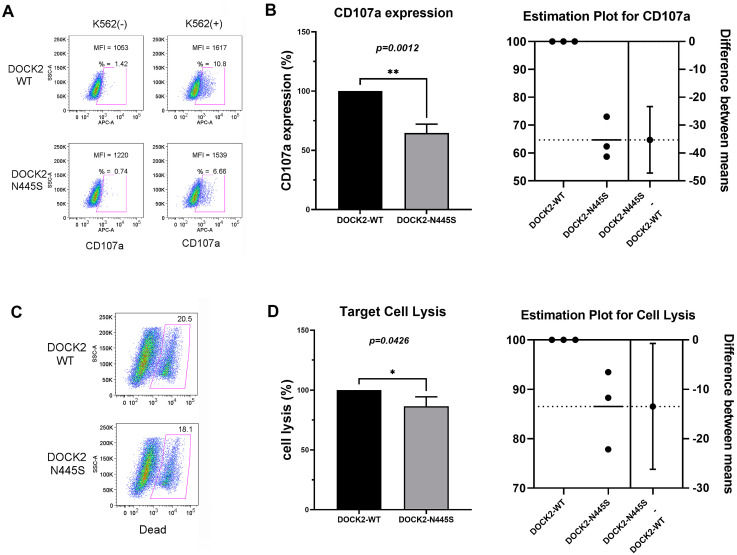
*DOCK2* p.Asn445Ser mutation decreased NK-cell cytotoxicity and degranulation. (**A**) CD107a expression was analyzed by flow cytometry in WT NK-92 cells and *DOCK2* p.Asn445Ser mutant NK-92 cells with and without K562 erythroleukemia cell co–stimulation. CD107a positive cells and mean fluorescence intensity are noted in each plot. (**B**) CD107a expression was significantly decreased in *DOCK2* p.Asn445Ser mutant NK-92 cells after two hours of stimulation with K562 cells as compared to WT (** = *p* < 0.01). (**C**) WT NK-92 and *DOCK2* p.Asn445Ser mutant NK-92 cells were mixed with K562 erythroleukemia cells for four hours at an effector-to-target cell ratio of 2:1 and stained with live/dead fixable cell dead reagent. Flow cytometry analysis of cell death is depicted along the x-axis. (**D**) *DOCK2* p.Asn445Ser mutant NK-92 cells caused statistically significantly less (* = *p* < 0.05) cell lysis when stimulated as compared to WT NK-92 cells. The pictured results in (**A**,**C**) are one representative example of the three independent experiments summarized in (**B**,**D**).

After lysosomal fusion with the cell membrane, CD107a cell surface expression occurs, and is, therefore, a reliable marker of NK cell degranulation [16]. At baseline and in the absence of K562 stimulatory cells, CD107a expression is low on both WT and mutant NK-92 cells (Figure 1A, left column). After NK-92 cell stimulation by K562 erythroleukemia cells, CD107a expression increased in both WT and *DOCK2* mutant NK-92 cells (Figure 1A, right column). However, the *DOCK2* mutant NK-92 cells exhibited significantly lower expression of CD107a, and therefore diminished degranulation capabilities than WT *DOCK2* over-expressing NK-92 cells. *DOCK2* mutant NK-92 cell CD107a expression was noted to be 65% of WT CD107a expression (*n* = 3, *p* = 0.0012), as seen in the estimation plot (Figure 1B).

The result of NK-cell degranulation and release of perforin and granzyme B is the lysis of pro-inflammatory target cells, ultimately calming the immune response. However, defects in this pathway can result in impaired cell lysis, leading to uncontrolled inflammation and HLH pathology. WT and *DOCK2* p.Asn445Ser mutant NK-92 cells were again mixed with K562 erythroleukemia cells, stimulating degranulation of the NK cells. Using live/dead fixable cell dead reagent, target cell lysis was measured by flow cytometry (Figure 1C). 86% as many target cells were lysed by *DOCK2* p.Asn445Ser mutant NK-92 cells as compared to WT NK-92 cells (*n* = 3, *p* = 0.426), meaning that the *DOCK2* p.Asn445Ser mutation resulted in significantly decreased cell lysis as compared to wild-type.

## 5. Discussion

### 5.1. DOCK2 and Immunodeficiency

Homozygous *DOCK2* deficiency has been implicated previously in immune dysfunction and immunodeficiencies. In a study of five patients with biallelic mutations in *DOCK2*, all were found to have invasive bacterial and viral infections early in life, and in vitro studies of patient immune cells showed multiple abnormalities [6]. Appropriate immune cell migration is important for trafficking cells to sites of infection and inflammation and in the development and maturation of lymphocytes. The DOCK2 protein has been shown to be influential in lymphocyte migration, chemotaxis, and actin cytoskeleton polymerization, and DOCK2 deficiency in mice results in both T and B lymphocyte migration abnormalities and T cell cytopenias [17]. In patients with *DOCK2* mutations, similar T cell lymphopenia has been seen, along with impaired lymphocyte actin cytoskeleton polymerization [6]. Another pathway disrupted by DOCK2 deficiency is the formation of human interferons (IFNs), essential signaling proteins in defense against viral infections. Type 1 IFN induction loss was found in DOCK2 deficient mice, and production of IFN-α and IFN-λ was decreased in three patients with *DOCK2* mutations [6,18]. Neutrophils can also be affected by DOCK2 deficiency. In a study of four siblings with a homozygous *DOCK2* mutation, all were noted to have impaired actin polymerization in neutrophils, impaired shape change (important in chemotaxis), and decreased production of reactive oxygen species [19].

### 5.2. DOCK2 and HLH

In this study, the patient’s own NK cells exhibited diminished NK cell lysis and degranulation ex vivo both during and in between HLH episodes when clinically well. In vitro, his rare heterozygous *DOCK2* mutation resulted in decreased NK cell degranulation and impaired cytotoxicity. This has been previously shown by Sakai et al., as although *DOCK2*^−/−^ NK cells can effectively bind to target cells in vivo, cytotoxicity and synapse formation are significantly diminished [7]. Similar findings were seen in a study of five patients with *DOCK2* mutations. These patients had normal circulating numbers of NK cells, but showed impaired degranulation after stimulation with human erythroleukemia K562 cells [6]. This impaired degranulation and cytotoxicity can lead to unchecked proliferation and pro-inflammatory activity of activated macrophages in patients with p- and sHLH. *DOCK2* mutations have been implicated in prior cases of HLH and severe infection. A 12-month-old patient with a *STAT2* pathogenic variant and unexplored heterozygous *DOCK2* mutation developed life-threatening HLH in the setting of recent one-year-old immunizations and human herpes virus type 6 reactivation, which spontaneously resolved [20]. This same patient was noted to have additional serious infections with influenza A, varicella-zoster activation after vaccination, and febrile seizures with seasonal coronavirus infection [20]. Another patient with DOCK2 deficiency developed HLH in the setting of Epstein-Barr virus infection after hematopoietic stem cell transplantation [21]. Finally, a variant in *DOCK2* was identified in a genome-wide association study for coronavirus disease-19 (COVID-19) in Japan and found to be associated with severe outcomes in patients under 65 years old [22]. In this study, postmortem lung samples of deceased COVID-19 patients were found to have decreased expression of DOCK2 in lung lymphocytes and macrophages, and inhibition of DOCK2 in a hamster model of COVID-19 resulted in increased severity of pulmonary edema, elevated viral loads, impaired macrophage response, and dysregulated IFN responses [22].

The rare missense *DOCK2* mutation (c.1334A>G, p.Asn445Ser) reported herein likely functions as a partial dominant-negative disrupting WT DOCK2 function in both the patient and in the NK-92 cell line with endogenous WT *DOCK2* expression where the *DOCK2* missense mutation is over-expressed. This defect resulting in baseline diminished NK cell cytolytic activity is likely tolerated until a sufficient level of immune hyper-activation is present. For this child with a *DOCK2* missense mutation, it was likely repeated episodes of bacterial sepsis that served as the triggering events. It is intriguing that during an sHLH episode, the child’s intracellular perforin and granzyme B levels were elevated, perhaps as a compensatory response to diminished NK cell lytic activity. Nonetheless, despite increased levels of perforin and granzyme B in the patient’s NK cells, NK cell dysfunction was still observed in the child with the *DOCK2* missense mutation.

Similar to DOCK2, the related DOCK180 family member, DOCK8, is important for NK cell function [23]. Recently, including in the setting of the hyper-inflammatory (sHLH-like) post-COVID-19 multi-system inflammatory syndrome in children (MIS-C), patient-derived *DOCK8* missense mutations have been shown to act in a partially dominant-negative manner to disrupt NK cell function [8,24]. Thus, both *DOCK2* and *DOCK8* missense mutations may contribute to sHLH in susceptible hosts by diminishing NK cell lytic function. Dominant-negative patient-derived missense mutations have been studied for several pHLH genes, including *PRF1*, *STXBP2*, and *RAB27A* [10,12,25,26,27,28,29]. While some missense variants in HLH genes can act as complete dominant-negative mutations, most have partial dominant-negative effects on perforin-mediated cytolysis, as is noted for the DOCK2 mutation reported herein [28]. In particular, the common *PRF1* p.Ala91Val variant has repeatedly demonstrated modest effects on perforin-mediated cytolysis [25,26]. While the complete absence of *PRF1* has been shown to result in prolonged engagement with the non-killed target cells yielding increased pro-inflammatory cytokines (e.g., IFN-γ) responsible for HLH, even partial disruption of perforin-mediated cytolysis by missense HLH gene mutations can delay cytolytic granule polarization yielding increased IFN-γ [29,30,31]. Interestingly, a parent with the same partial dominant-negative missense mutation in *RAB27A* contributing to CSS in his child has not experienced CSS despite having a similar decreased NK cell function as his child at baseline [29]. This can be explained using a threshold model of HLH disease development, such that genetically determined baseline partially disrupted NK cell dysfunction is tolerated until an insult (frequently infectious) overwhelms the immune regulatory system such that clinical HLH is evident [29,32,33]. Thus, partial dominant-negative mutations in *DOCK2* (herein) and *DOCK8* altering NK cell lytic function may contribute to sHLH development during excess inflammatory states such as sepsis [8,34].

## 6. Conclusions

HLH is a serious medical condition with the potential to cause severe morbidity and mortality in the pediatric population. DOCK2 and the related DOCK180 family protein, DOCK8, are increasingly recognized as important regulators of immune function, including NK cell cytolytic activity. Identification of sHLH patient-derived partial dominant-negative missense mutations in both *DOCK2* (herein) and *DOCK8* suggest their contributions to HLH pathogenesis. As genetic testing becomes more readily available at the bedside, with improving turn-around times, clinicians can identify patients at risk for primary and secondary HLH like never before. Still needed are larger pediatric population-based studies to identify patients with both known and novel mutations in HLH-related genes, combined with confirmatory laboratory investigation of the effects of the patient-derived mutations on HLH pathogenesis. With an increased understanding of HLH genetics, clinicians will have the opportunity to improve HLH diagnosis and treatment to mitigate the risk of serious morbidity and mortality.

## Figures and Tables

**Table 1:** Hospitalizations and blood counts.

Age (Years)	Admission	WBC Count	ANC	ALC	Hgb	Platelet Count
		×10^3^/μL	×10^3^/μL	×10^3^/μL	g/dL	×10^3^/μL
7	Fever, emesis Negative infectious workup	Min: **1.53** Max: 7.24 (nml 4.31–11.0)	Min: **0.72** (nml 1.63–7.55)	Min: **0.34** (nml 0.97–3.96)	Min: **6.5** (nml 10.7–13.4)	Min: **108** Max: 426 (nml 140–440)
7	Positive blood cultures: *Staphylococcus aureus* *Candida albicans*	Min: **1.82** Max: 8.57 (4.31–11.0)	Min: **1.09** (1.63–7.55)	Min: **0.43** (0.97–3.96)	Min: **6.3** (10.7–13.4)	Min: **55** Max: **471** (140–440)
7	Fever, cough, congestion Negative infectious workup	Min: **1.73** Max: 6.55 (4.31–11.0)	Min: **1.28** (1.63–7.55)	Min: **0.19** (0.97–3.96)	Min: **9.2** (10.7–13.4)	Min: **90** Max: 277 (140–440)
8	Positive blood cultures: *Candida albicans*	Min: **2.90** Max: 9.09 (4.31–11.0)	Min: **1.61** (1.63–7.55)	Min: **0.42** (0.97–3.96)	Min: **6.3** (10.7–13.4)	Min: **34** Max: **112** (140–440)
8	Fever, lethargy Negative infectious workup	Min: **0.39** Max: **2.47** (4.31–11.0)	Min: **0.23** (1.63–7.55)	Min: **0.14** (0.97–3.96)	Min: **6.9** (10.7–13.4)	Min: **94** Max: 400 (140–440)
10	Positive blood cultures: *Bacillus licheniformis*	Min: **0.73** Max: 4.82 (4.31–11.0)	Min: **0.45** (1.63–7.55)	Min: **0.24** (0.97–3.96)	Min: **5.8** (10.7–13.4)	Min: **25** Max: 394 (140–440)
10	Fever, lethargy Negative infectious workup	Min: **1.05** Max: **2.86** (4.31–11.0)	Min: **0.61** (1.63–7.55)	Min: **0.41** (0.97–3.96)	Min: **6.4** (10.7–13.4)	Min: **63** Max: 155 (140–440)
10	Positive blood cultures: *Staphylococcus aureus*	Min: **2.72** Max: 5.01 (4.31–11.0)	Min: 1.97 (1.63–7.55)	Min: **0.44** (0.97–3.96)	Min: **8.8** (10.7–13.4)	Min: **40** Max: **124** (140–440)
10	Positive blood cultures: *Staphylococcus aureus*	Min: **0.96** Max: **1.07** (4.31–11.0)	Min: **0.76** (1.63–7.55)	Min: **0.26** (0.97–3.96)	Min: **8.1** (10.7–13.4)	Min: **197** Max: 236 (140–440)
11	Fever, lethargy Negative infectious workup	Min: **2.40** Max: **3.81** (4.31–11.0)	Min: **1.26** (1.63–7.55)	Min: **0.59** (0.97–3.96)	Min: **9.6** (10.7–13.4)	Min: **109** Max: 262 (140–440)
11	Positive blood cultures: *Klebsiella pneumoniae*, *Staphylococcus aureus*	Min: **1.98** Max: 5.93 (4.31–11.0)	Min: **1.04** (1.63–7.55)	Min: **0.26** (0.97–3.96)	Min: **7.5** (10.7–13.4)	Min: **51** Max: 239 (140–440)

Values outside of the normal ranges are bolded. Abbreviations: WBC—white blood cell; ANC—absolute neutrophil count; ALC—absolute lymphocyte count; Hgb—hemoglobin; nml—normal; min—minimum value; max—maximum value.

**Table 2:** Hospitalizations and inflammatory/liver markers.

Age (Years)	Admission	AST	ALT	Ferritin	CRP	ESR	Fibrinogen	LDH
		U/L	U/L	ng/mL	mg/dL	mm/hr	mg/dL	U/L
7	Fever, emesis Negative infectious workup	Max: **399** (nml 15–40)	Max: **104** (nml 10–35)	Max: **52,650** (nml 22–340)	Max: **6.61** (nml < 0.5)	Min: 6 Max: **76** (nml 0–15)	Max/Min: 323 (nml 156–400)	Max: **10,884** (nml 420–750)
7	Positive blood cultures: *Staphylococcus aureus* *Candida albicans*	Max: **392** (15–40)	Max: **118** (10–35)	Max: **36,972** (22–340)	Max: **21.12** (<0.5)	Min: 11 Max: **73** (0–15)	Min: **128** Max: **559** (156–400)	–
7	Fever, cough, congestion Negative infectious workup	Max: **229** (15–40)	Max: **316** (10–35)	Max: **13,194** (22–340)	Max: **8.84** (<0.5)	Min: 7 Max: **16** (0–15)	Max/Min: 195 (nml 156–400)	Max: **4272** (420–750)
8	Positive blood cultures: *Candida albicans*	Max: **76** (18–36)	Max: **45.5** (9.0–25.0)	Max: **1957** (13.7–78.8)	Max: **13.23** (<0.5)	Min: 14 Max: **52** (0–15)	Min: 309 Max: **532** (156–400)	–
8	Fever, lethargy Negative infectious workup	Max: **175** (18–36)	Max: **72.5** (9.0–25.0)	Max: **20,455.6** (13.7–78.8)	Max: **3.6** (<0.5)	Min: 11 Max: **30** (0–15)	–	–
10	Positive blood cultures: *Bacillus licheniformis*	Max: **127** (18–36)	Max: **121.3** (9.0–25.0)	Max: **4429.6** (13.7–78.8)	Max: **7.88** (<0.5)	Min: 9 Max: **30** (0–15)	Min: **84** Max: 197 (156–400)	Max: **1630** (170–283)
10	Fever, lethargy Negative infectious workup	Max: **225** (18–36)	Max: **168.9** (9.0–25.0)	Max: **2727.1** (13.7–78.8)	Max: **4.42** (<0.5)	Min: 9 Max: **24** (0–15)	Max/Min: 160 (nml 156–400)	–
10	Positive blood cultures: *Staphylococcus aureus*	Max: **100** (18–36)	Max: **100** (9.0–25.0)	Max: **896.1** (13.7–78.8)	Max: **15.52** (<0.5)	Min: **33** Max: **51** (0–15)	–	–
10	Positive blood cultures: *Staphylococcus aureus*	Max: **171** (18–36)	Max: **134.5** (9.0–25.0)	Max: **827.1** (13.7–78.8)	Max: **1.88** (<0.5)	Min: 15 Max: **28** (0–15)	–	–
11	Fever, lethargy Negative infectious workup	Max: **95** (18–36)	Max: **83.5** (9.0–25.0)	Max: **239.1** (13.7–78.8)	Max: **13.46** (<0.5)	Min/Max: 13 (0–15)	–	–
11	Positive blood cultures: *Klebsiella pneumoniae*, *Staphylococcus aureus*	Max: **44** (18–36)	Max: **39** (9.0–25.0)	Max: **196** (13.7–78.8)	Max: **14.3** (<0.5)	Min: **48** Max: **77** (0–15)	–	Max: 241 (170–283)

Values outside of the normal ranges are bolded. Abbreviations: AST—aspartate aminotransferase; ALT—alanine transaminase; CRP—c-reactive protein; ESR—erythrocyte sedimentation rate; LDH—lactate dehydrogenase; nml—normal; min—minimum value; max—maximum value.

**Table 3:** Immunodeficiency genetic panel results.

Gene	Variant	Zygosity	Variant Classification	Disease Association
G6PD	c.[202G>A;376A>G] (p.[Val68Met;ASn126Asp])	hemizygous	Pathogenic	X-linked G6PD deficiency
PEPD	Deletion (Exon 1)	heterozygous	Pathogenic	Autosomal recessive prolidase deficiency
PEPD	c.932G>A (p.Arg311Gln)	heterozygous	Uncertain Significance	Autosomal recessive prolidase deficiency
DOCK2	c.1334A>G (p.Asn445Ser)	heterozygous	Uncertain Significance	Autosomal recessive combined immunodeficiency due to DOCK2 deficiency
ERCC6L2	c.4089T>G (p.Asn1363Lys)	heterozygous	Uncertain Significance	Autosomal recessive ERCC6L2 deficiency
FANCA	c.753_755delinsAG (p.Asp252Ser)	heterozygous	Uncertain Significance	Autosomal recessive Fanconi anemia type A
FCHO1	c.529C>T (p.Arg177Cys)	heterozygous	Uncertain Significance	Autosomal recessive combined immunodeficiency due to FCHO1 deficiency
G6PC3	c.50A>C (p.Asn17Thr)	heterozygous	Uncertain Significance	Autosomal recessive severe congenital neutropenia
RBCK1	c.1028C>T (p.Ala343Val)	heterozygous	Uncertain Significance	Autosomal recessive polyglucosan body myopathy with or without immunodeficiency
RTEL1	c.3499+7C>T (intronic)	heterozygous	Uncertain Significance	Autosomal recessive dyskeratosis congenital spectrum disorders
SAMD9L	c.251dup (p.Asn84Lysfs * 3)	heterozygous	Uncertain Significance	Autosomal dominant ataxia-pancytopenia syndrome
UNC45A	c.2003C>A (p.Ser668Tr)	heterozygous	Uncertain Significance	No well-established disease association

## Data Availability

Data available upon request to the corresponding author.

## Supplementary Materials

The following supporting information can be downloaded at: https://www.mdpi.com/article/10.3390/life13020434/s1, Table S1. Negative HLH genes.
