# Supplementary material for: DOCK2 Mutation and Recurrent Hemophagocytic Lymphohistiocytosis
Source: Life (Basel). 2023 Feb 3;13(2):434. doi: 10.3390/life13020434 (PMC9962445; doi:10.3390/life13020434)
Supplement: Supplementary file 1 [file life-13-00434-s001.zip › life-2150147-supplementary.pdf]

Supplemental Table S1. Negative HLH genes

Gene	Associated Conditions
ADA	Adenosine deaminase deficiency
AP3B1	Hermansky-Pudlak syndrome type 2
BLOC1S6	Hermansky-Pudlak syndrome type 9
BTK	X-linked agammaglobulinemia
CD27	Lymphoproliferative syndrome type 2
IL2RA	IL2RA (CD25) deficiency<br>Immunodeficiency 41 with lymphoproliferation and autoimmunity
IL2RG	X-linked severe combined immunodeficiency (X-SCID)
ITK	Lymphoproliferative syndrome type 1
LYST	Chediak-Higashi Syndrome
MAGT1	X-linked immunodeficiency with magnesium defect<br>Epstein-Barr virus infection and neoplasia (XMEN)
MVK	Mevolanate kinase deficiency
PNP	Purine nucleoside phosphorylase deficiency
PRF1	Familial hemophagocytic lymphohistiocytosis type 2
RAB27A	Griscelli syndrome type 2
SH2D1A	X-linked lymphoproliferative disease type 1
SLC7A7	Lysinuric protein intolerance
STX11	Familial hemophagocytic lymphohistiocytosis type 4
STXBP2	Familial hemophagocytic lymphohistiocytosis type 5
UNC13D	Familial hemophagocytic lymphohistiocytosis type 3
WAS	Wiskott-Aldrich syndrome
XIAP	X-linked lymphoproliferative disease type 2
